# Factors Associated with Medication Adherence in Elderly Individuals with Tuberculosis: A Qualitative Study

**DOI:** 10.1155/2023/4056548

**Published:** 2023-03-08

**Authors:** Somayeh Hassani, Farahnaz Mohammadi Shahboulagi, Mahshid Foroughan, Seyed Alireza Nadji, Payam Tabarsi, Gholamreza Ghaedamini Harouni

**Affiliations:** ^1^Iranian Research Center on Aging, University of Social Welfare and Rehabilitation Sciences (USWR), Tehran, Iran; ^2^Iranian Research Center on Aging, Nursing Department, University of Social Welfare and Rehabilitation Sciences, Tehran, Iran; ^3^Virology Research Center, National Research Institute of Tuberculosis and Lung Diseases (NRITLD), Shahid Beheshti University of Medical Sciences, Tehran, Iran; ^4^Clinical Tuberculosis and Epidemiology Research Center, National Research Institute of Tuberculosis and Lung Diseases (NRITLD), Shahid Beheshti University of Medical Sciences, Tehran, Iran; ^5^Social Welfare Management Research Center, University of Social Welfare and Rehabilitation Sciences (USWR), Tehran, Iran

## Abstract

**Methods:**

This qualitative study was conducted in two phases, using an integrative literature review and individual interviews. Studies were gathered without time restriction from MEDLINE databases, Institute for Scientific Information (ISI), Google Scholar, Scopus, and EMBASE, as well as national databases, including Scientific Information Database and Magiran. The findings of 38 studies that met the inclusion criteria were analyzed through the conventional content analysis method based on the ecological approach. After reviewing and forming the data matrix, purposive sampling was performed among healthcare professionals, elderly tuberculosis patients aged 60 and over, and family caregivers of elderly patients to conduct individual interviews. Data obtained from 20 interviews were analyzed using the directed content analysis method. After coding, the data from individual interviews were entered based on similarity and difference in the categories of data matrix obtained from the literature review.

**Results:**

In general, the aforementioned codes were placed in four main categories, including individual factors (i.e., biological factors, affective-emotional factors, behavioral factors, cognitive factors, tuberculosis-related factors, and economic factors), interpersonal factors (i.e., patient's relationship with treatment team and family-related factors), factors related to healthcare service provider centers (i.e., medical centers' facilities and capacity building in healthcare service provider), and extraorganizational factors (i.e., social factors and health policymaking).

**Conclusion:**

The results of this study showed that medication adherence in elderly patients with tuberculosis was a complex and multidimensional phenomenon. Therefore, society, policymakers, and healthcare providers should scrutinize the factors affecting medication adherence in this group of patients to plan and implement more effective interventions.

## 1. Introduction

One of the most important ways to control and eradicate tuberculosis is successful treatment [1], and the main determinant in a successful treatment is medication adherence [2–7]. Despite various interventions, such as directly observed treatment [4, 8], tuberculosis medication nonadherence still occurs [9, 10] and a significant number of tuberculosis patients do not have appropriate medication adherence [2, 11]. Tuberculosis specialists have estimated the spectrum of medication nonadherence to be 20–100% [4].

Medication nonadherence in tuberculosis reduces the rate of successful treatment [12] and recovery [13]. In addition, it causes failure in treatment, prolonged hospitalization, and delay in treatment completion, and as a result of prolonged treatment process, there will be increased treatment costs, psychological complications [2, 14], recurrence of the disease, increased risk of drug resistance, and increased death rates related to disease [8, 11, 14–17]. Each patient with active tuberculosis causes the infection of 5–15 individuals in a year [18], and failure in the treatment and prolongation of the treatment process will, in turn, lead to the increased likelihood of tuberculosis prevalence in society and the increased burden of the disease [6, 11, 14, 19].

Some studies have shown that nonadherence [20], poor adherence, and default treatment process are more common in elderly tuberculosis patients [21, 22] to the extent that some scientific evidences consider that the failure in eradicating tuberculosis results from treatment failure in the elderly group [8, 23, 24].

The global frequency of tuberculosis in the elderly is three times [21, 25], and related death rate is six times higher than nonelderly individuals [26]. It is expected that following demographic changes and the increase in number of elderly individuals, the incidence of tuberculosis will also increase in this age group [21]. As a result, the tuberculosis control in the elderly can affect tuberculosis prevention and control programs in all countries [25, 27].

Given the importance of medication adherence in tuberculosis, especially in elderly patients, it is necessary to recognize the factors related to medication adherence and investigate them to empower tuberculosis eradication strategies [28]. Identifying these factors and carrying out appropriate interventions can improve medication adherence and subsequently that lead to more effective tuberculosis control [29] and help policymakers, healthcare providers, society, and patients in solving tuberculosis-related problems [30].

Although numerous studies have been conducted about the factors associated with medication adherence in tuberculosis patients, there are few studies specifically in the field of the elderly [31, 32]. Since disease manifestations, diagnoses, consequences, and treatments in the elderly are different from other age groups [27, 33, 34], it is necessary to consider the factors related to medication adherence in this group of patients separately.

Treatment adherence behavior is a complex and dynamic phenomenon and is related to a wide range of geographic, cultural [19], social, and economic factors [35, 36]. Treatment adherence is not merely a medical phenomenon, and related psychological and social dimensions can be expected. Understanding such phenomena requires a holistic approach that can be achieved through qualitative studies. The recent interest in the studies related to the qualitative approach in the field of medication adherence confirms this important issue. Qualitative studies and paying attention to the opinions, perceptions, and experiences of patients and groups related to tuberculosis treatment lead to a better and deeper understanding of these factors [35, 37] based on the context of a tuberculosis patient's life and the healthcare service provider system [38, 39], better understanding and interpretation of findings obtained from quantitative studies [11], clarifying the medication adherence phenomenon [40–42], and as a result facilitating interventions, empowering patients, and improving medication adherence [35].

According to the abovementioned conditions, the knowledge about factors affecting the medication adherence of elderly tuberculosis patients is limited, and qualitative studies play an important role in identifying and deeply understanding these factors. Therefore, the aim of this study was explaining the factors related to medication adherence based on the healthcare professionals, elderly tuberculosis patients, and their caregivers' experiences.

## 2. Methods

### 2.1. Study Design, Sample Size, and Sample Collection

The present study consisted of two parts. First, an integrative literature review [43] was carried out, and the data were analyzed based on conventional content analysis [44]. The second part of the study was conducted based on the interviews with healthcare professionals (i.e., physicians and nurses), elderly tuberculosis patients, and family caregivers of the elderly. Furthermore, the directed content analysis method was used to analyze the data obtained from the interviews [44].

In the integrative literature review section, the literature search was conducted in November 2020 according to systematic review studies and without time restrictions. The titles and abstracts of English and Persian articles were used using the keywords, including adherence, nonadherence, treatment nonadherence, medication adherence, medication nonadherence, concordance, tuberculosis, elderly, older individuals, aging, and old were used individually, in addition to MEDLINE databases (including PubMed and Ovid), Institute for Scientific Information (ISI), Google Scholar, Scopus, EMBASE, and national databases, including Scientific Information Database, Magiran, as well as national journals.

**Figure 1:**
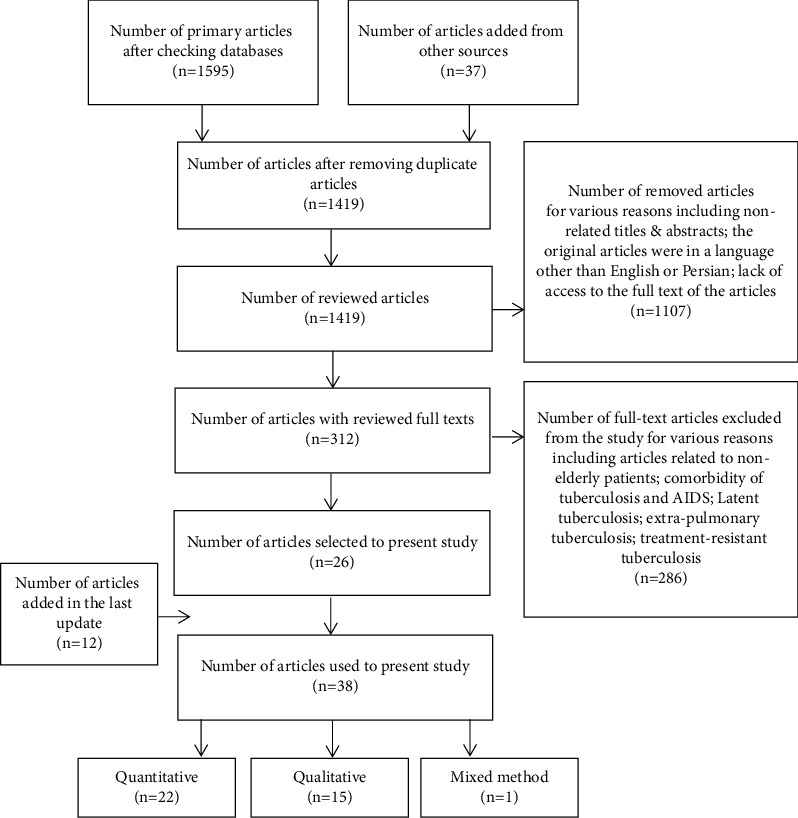
Article selection process.

In addition, a manual search was performed by reviewing the references of the selected articles, and related articles were retrieved. Full-text research articles on medication adherence in tuberculosis (with quantitative and qualitative methods) were included in the study. First, the titles of the articles were examined, and duplicate articles were excluded from the study. Among the remaining articles, the abstracts of the articles that met inclusion criteria were independently reviewed by two members of the research team. Finally, 38 studies were analyzed. The mixed methods appraisal tool (MMAT) (version 2018) was used to check the quality of the studies [45–50]. Based on the MMAT, all the studies had suitable quality to be included in the review. The article selection process was implemented based on the PRISMA protocol (Figure 1).

In the second part, after compiling the data analysis matrix obtained from the integrative literature review, individual interviews were conducted with the participants. The participants were selected by purposive sampling including the healthcare professionals (i.e., physicians and nurses) who had the experience of working with tuberculosis patients; elderly tuberculosis patients who aged 60 and over with a definite diagnosis of pulmonary tuberculosis, diagnosis as a new patient, passing at least one month since the start of treatment, the ability to communicate, no cognitive problem (according to the abbreviated mental test) [51], and referring to Dr. Masih Daneshvari Hospital clinic (As a reference center for tuberculosis in Iran) and health centers covered by Shahid Beheshti University of Medical Sciences, Tehran, Iran; as well as family caregivers of the elderly who were partially or fully responsible for caring an elderly TB patient for at least 6 months.

Individual and semistructured interviews were conducted during April and October 2021. After inviting the participants to attend the interviews and assuring them of information confidentiality, written informed consent was obtained. The interviews were conducted in a private room at the infectious disease clinic or the patient's home or workplace. At the time of conducting the interview, the interview process was recorded with the subjects' permission. The interviews continued until data saturation was reached. The healthcare professionals had to answer the questions, such as “In your opinion, what individual, interpersonal, treatment system, and social factors can affect the use of medications in elderly tuberculosis patients?.” Questions related to the patients and caregivers were designed more simply, for example patient related questions such as “Has it ever happened that you did not take your medicines as prescribed by the physician? Why?” Or “Can you explain what factors make it difficult for you to take medicines?;” besides a question such as “Is your patient facing a problem in taking his/her medications? Please explain.” Was asked from their caregivers. The interviews continued with asking follow-up and exploratory questions. 20 interviews with the participants were conducted and analyzed (Table 1).

### 2.2. Ethical Issues

The present study was a part of a PhD thesis and was approved by the Ethics Committee of the University of Rehabilitation Sciences and Social Health, Tehran, Iran. Necessary permits to conduct the research were obtained from the vice-chancellor of Research. All the subjects were free to participate or leave the study. In all stages of the project implementation, the principle of information confidentiality of the participants was observed.

### 2.3. Data Analysis

In the integrative literature review section, conventional content analysis with the Elo and Kyngäs method (2008) was used for data analysis [44]. The researcher used the ecological approach in this section to achieve a comprehensive view [52–55]. First, the findings of the articles were examined, and the factors affecting medication adherence were extracted. Then, data coding and factor classification were performed. The analysis of this part of the study was used to produce the data analysis matrix of the second part.

In the second part of the study, after conducting each interview, the audio file of the interview was listened several times. Finally, the interview transcript was prepared in a typed form. Simultaneously with the progress of the interviews, data analysis was performed with the directed content analysis method through the Elo and Kyngäs method [44]. For this purpose, the text of each interview was read several times to immerse in the data and gain a general understanding of the interview. Then, coding was done and the codes obtained from each interview were entered based on similarities and differences in the categories of the data matrix obtained from the literature review. The codes that were not included in any of the categories of the data matrix formed new categories and subcategories using the inductive approach.

## 3. Results

In the first part of the study, based on the ecological approach, the codes extracted from the studies were placed in three main categories, including individual, interpersonal, and extraorganizational factors. The codes obtained from the interviews with the participants were also placed based on similarities and differences in the categories of the matrix obtained from the literature review. The codes that were not included in any of the categories of the data matrix formed new categories and subcategories using the inductive approach as follows (shown in gray in Table 2):

Primary subcategories, including the polypharmacy, activities of daily living (ADL), instrumental activities of daily living (IADL), patient's personality, acceptance of illness, professional competence of the treatment team, educating the patient and caregiver by the treatment team, general characteristics of the family caregiver and family caregiver knowledge, and the main category of factors related to healthcare service provider centers.

In general, the codes obtained from the integrative literature review and individual interviews formed four main categories, including individual factors, interpersonal factors, factors related to healthcare service provider centers, and extraorganizational factors (Table 2).

### 3.1. Category of Individual Factors

The main category of individual factors was obtained from the subcategories of biological factors, affective-emotional factors, behavioral factors, cognitive factors, tuberculosis-related factors, and economic factors. Most of the codes extracted from the integrative literature review and individual interviews were placed in this category.

The subcategory of biological factors dealt with the individual characteristics affecting the medication adherence of the elderly tuberculosis patients. The analysis of the data obtained from the literature review confirmed that the age [20, 31, 32, 56–66], gender [22, 61, 66], and comorbidity [21, 57, 60, 67, 68] affected the medication adherence of elderly tuberculosis patients. The interviews also confirmed the importance of these factors and their impact on medication adherence. In addition, the participants' experiences in the interviews indicated the impact of the polypharmacy, activities of daily living (ADL), and instrumental activities of daily living (IADL) on the medication adherence behavior of elderly tuberculosis patients.

Regarding age, the results of the studies were contradictory; however, most studies showed decreased medication adherence by age increasing [31, 56–59]. The interviews with healthcare professionals also indicated less adherence in older ages. The results of the review regarding the effect of gender on the medication adherence of elderly patients with tuberculosis were also contradictory; nevertheless, most studies demonstrated lower adherence in male patients [22, 61]. A nurse with work experience in teaching patients about comorbidity and polypharmacy said:

*“…Undoubtedly, the elderly do not take a large number of medicines and pills, or for example, they have problems, such as advanced rheumatoid arthritis; it is even difficult for them to take the pills out of their covers, or they have vision problems and cannot see their medicine well.”*

The patients believe that the ability to perform activities of daily living and instrumental activities of daily living is an important factor for tuberculosis medication adherence. In this regard, a 76-year-old female patient said:

*“I'm happy that God has given me the physical ability, and I am able to do the treatment affairs and take medicines as much as possible.”*

The subcategory of affective-emotional factors referred to affective and emotional factors. The analysis of data obtained from the literature review confirmed that motivation for treatment [69, 70], depression and anxiety [70, 71], and patient's affections and feelings [66] affected the medication adherence of elderly tuberculosis patients. The participants' experiences in the interviews indicated the impact of the patient's personality, and acceptance of illness on medication adherence behavior of elderly tuberculosis patients. The 76-year-old female patient said:

*“…I believed that I should take medicine to get better and not be a burden on my children. My illness was a great sorrow for them and this hurt me.”*

The subcategory of behavioral factors referred to the patient's self-care ability [57, 70], substance abuse [66, 69, 70], and the use of reminders for taking medication [72, 73] based on the literature review and participants' experiences in the interviews. An 86-year-old male patient said:

*“…Every month, the physician gave me pills, and I was supposed to take the pills. I never left the house and I always took my medicine on time. A wise person should stick to the physician's orders.”*

The subcategory of cognitive factors referred to the analysis of data obtained from the literature review insisted on effective interaction with the physician [74, 75], patient's memory [60], patient's knowledge about tuberculosis and treatment [67, 69, 70, 74, 76–80], and patient 's general knowledge [59, 66, 81]. The interviews also confirmed the importance of these factors and their impact on medication adherence in elderly tuberculosis patients. One of the physicians in this regard said:

*…“The effective interaction of the patient with the physician and the correct understanding of the physician's explanations about taking the medications are very important. We had elderly patients who understood the detailed explanations we gave them very well and cooperated with us.”*

One of the physicians, regarding the patient's knowledge about tuberculosis and treatment, said:

*…“When a patient gets tuberculosis, he/she should understand the danger for him/herself and society. When the patient understands, he/she changes his/her behavior to escape from this danger and eliminates this threat. The first change in his/her behavior is the correct consumption of medications.”*

The results of studies on the effect of the patient's general knowledge and education on the medication adherence of elderly tuberculosis patients were contradictory [59, 66, 80]. In this regard, the majority of healthcare professionals believed that the education of the elderly affected tuberculosis medication adherence.

The subcategory of factors related to the disease and antituberculosis treatment referred to the type of tuberculosis [21, 22, 61], characteristics of pharmacotherapy [57, 66], response to treatment [70, 75], and medication side effects [58, 67, 70] based on the literature review and participants' experiences in the interviews. The prolonged tuberculosis treatment course is one of the factors affecting tuberculosis medication nonadherence [66]. One of the physicians said:

*…“The elderly themselves might say that nothing has left until the end of my life. Six months of taking tuberculosis medications seem like a very long time to them.”*

A 67-year-old male patient about the effect of treatment response said:

*…“I took medicine for a while, but I do not feel sick anymore, and I think I do not need medicine anymore.”*

The results of this study showed that the management of side effects of the patients was effective in the treatment continuation. In this regard, a 67-year-old female patient said:

*…“I could not take medicine, and I stopped it. I came to the hospital; they told me I should be admitted to see why the medicine bothered me. I was hospitalized for 12 days, and they gave me antiallergic medicine. Afterward, the tuberculosis medicines did not bother me anymore, and I took all the medicines.”*

The subcategory of economic factors referred to economic issues. The analysis of the data obtained from the literature review and interviews confirmed that patient's financial ability [78, 81] and the patient's employment [67, 74, 82] impact on medication adherence in elderly individual with tuberculosis. One of the family caregivers in this regard said:

*…“For a patient who does not have money, taking medicine does not make sense. He/she says that I do not have money, so I will not take medicine until I die.”*

A 72-year-old male patient in this regard said:

*…“I am a simple daily wage worker. Due to tuberculosis, I can no longer do heavy work. I only go for light work. I hardly come to the hospital due to my job and financial problems.”*

### 3.2. Category of Interpersonal Factors

The main category of interpersonal factors was obtained from the subcategories of relationship with the treatment team and family-related factors.

The subcategory of relationship with the treatment team referred to the professional ethics of the treatment team [67, 74, 82] and trust in the treatment team [70, 82] according to the available evidence based on the literature review. In addition to the abovementioned cases, the participants in the interviews emphasized the impact of the professional competence of the treatment team and educating the patient and caregiver by the treatment team. One of the essential codes extracted in the field of professional ethics of the treatment team, which was mentioned in the literature review and interviews, was the establishment of proper communication between the treatment team and the patient [82]. One of the nurses in this regard said:

*…“I think it is crucial for the treatment team to accept the patient as a human being and establish a proper relationship with him/her.”*

A 76-year-old female patient in this regard said:

*…“My physician was very good. While he is paying attention to other patients, he also pays attention to me and answers my questions.”*

One of the pulmonologists about the professional competence of the treatment team said:

*…“After 24 years of work experience, I've found out whether the patient really had the necessary capacity to take the medicines or not and understand my training or needed more explanations and follow-up.”*

A 76-year-old male patient about trust in the treatment team said:

*…“I had an argument with a nurse in the clinic. The same nurse came to the ward and did not let the physician examine me. I realized that the nurse had enmity with me, and I would not get better in that hospital. So, I left the hospital and discontinued my treatment.”*

One of the physicians about the importance of educating the patient said:

*…“It happens many times that a family member comes and insists that he/she must take this medicine by him/herself and deliver it to the patient. Then, we have to see if the caregiver has received the necessary training and transfer this training (regarding how to take medicine, medicine side effects, and when to come and let us know if the medicine causes complications) to the elderly in the correct way. All these are some of the problems we have in the treatment of elderly patients with tuberculosis.”*

The subcategory of family-related factors it refers to the characteristics of the patients' family members. The review of studies showed the effect of the presence of a supportive family [66, 69, 70, 74] on the medication adherence of elderly tuberculosis patients. The experiences of the participants in the interviews showed that not only the supportive characteristics but also the family caregiver's general characteristics and knowledge influence the medication adherence of this group of tuberculosis patients. An 86-year-old male patient in this regard said:

*…“Now, I cannot go to the reception and take medicine myself anymore. My children do all the affairs related to my medicine and treatment.”*

A 67-year-old patient said:

*…“From the beginning of the treatment, my wife was very concerned about taking the medicines on time. She set the alarm and gave me medicine at the exact time when I should have taken medicine.”*

One of the physicians said about family caregiver's knowledge:

*…“An elderly patient with tuberculosis needs a well-educated supervisor for his/her treatment. Unfortunately, we are often weak in this regard.”*

Regarding the general characteristics of a family caregiver, one of the caregivers stated:

*…“All my siblings are employed and married and we cannot fully supervise my father's medication intake, so sometimes my father does not take his medications.”*

### 3.3. Category of Factors Related to Healthcare Service Provider Centers

This category was obtained using an inductive approach from the subcategories of medical centers' facilities and capacity building in healthcare service provider.

The subcategory of medical centers' facilities referred to responding to the patients' needs, centers' equipment, and resource adequacy. No study was identified related to the aforementioned subcategory, and this subcategory was created based on the participants' experiences. One of the physicians in this regard said:

*…“Our centers are very crowded. The elderly do not have the patience to sit in a long queue and they might not be referred to our centers at all.”*

Another physician suggested:

*…“There should be special days to visit elderly tuberculosis patients. In this case, the physician or the relevant expert will examine the patient at ease. A tuberculosis patient is a time-consuming patient. On the other hand, an elderly patient does not understand the content and is not satisfied by explaining once.”*

Some caregivers pointed to the lack of proper response of medical centers following the coronavirus disease 2019 (COVID-19) pandemic. One of the caregivers said in this regard:

*…“The main problem is making an appointment for visits and receiving medicines. After the onset of the COVID-19 pandemic, it has become very difficult to make an appointment.”*

One of the pulmonologists about the resource adequacy said:

*…“Currently, when we are dealing with COVID-19, unfortunately, chronic and infectious diseases, such as tuberculosis, are on the sidelines because our health and treatment resources are limited.”*

A 67-year-old male patient also in this regard said:

*…“I referred to the clinic to receive medicine, but this month, the medicine had not come due to the COVID-19 pandemic. Therefore, I stopped taking my medicines for 2 days.”*

The subcategory of capacity building in healthcare service provider was also created only based on the codes obtained from interviews with the participants. This subcategory referred to educating the staff, monitoring the health of the staff, and motivating the staff. In this regard one of the physicians said:

*…“In order to achieve the desired result in the treatment of tuberculosis, we should increase the awareness of the staff regarding the issues of the elderly patients.”*

Another physician suggested:

*…“In order to be successful in the tuberculosis treatment, we need to take care of both physicians and nurses and have special privileges to work in the wards related to tuberculosis.”*

### 3.4. Category of Extraorganizational Factors

The main category of extraorganizational factors was obtained from the subcategories of social factors and health policymaking.

The subcategory of social factors referred to the tuberculosis stigma [37, 67, 69, 70, 75, 80, 83], belief in traditional treatment [79], social development [19, 66, 74, 84], and social support [69, 70, 74] according to the available evidence based on the literature review. The participants' experiences in the interviews also emphasized the aforementioned items. One of the physicians about stigma said:

*…“The tuberculosis stigma leads to the rejection of the patient by the family, causes emotional and mental problems, and affects taking medications.”*

One of the caregivers also in this regard said:

*…“I did not tell a word the people around me about my husband's tuberculosis. Anyone who asked, we said that he had a cold and his lungs were infected. If we say that he has tuberculosis, people's behavior will change. People will run away from him, and as a result, his mood will deteriorate, and he might not be able to continue treatment.”*

One of the caregivers about taking traditional medications said:

*…“I used herbal medicines to relieve my father's pain and cough. Finally, this forced me to take him to the hospital and caused delayed treatment.”*

One of the nurses about social development said:

*…“It is difficult for the elderly to go to health centers. Some elderly individuals have to use the bus to reach the hospital. Due to the problems of public transportation, the elderly patients might often not refer to the health centers.”*

One of the physicians, regarding the effect of place of residence on medication adherence, said:

*…“Elderly individuals with tuberculosis who live in urban areas have better medication adherence.”*

The subcategory of health policymaking based on the evidence from the literature review referred to the existing treatment protocols [61, 75, 76, 85] and the level of insurance coverage [66, 67, 81]. The participants' experiences also confirmed the aforementioned issue. One of the physicians in this regard said:

*…“In my opinion, the implementation of directly observed treatment is critical and effective in the medication adherence of elderly patients with tuberculosis.”*

One of the pulmonologists in this regard said:

*…“COVID-19 pandemic has affected tuberculosis treatment policies. Tuberculosis diagnosis has decreased. Patients with tuberculosis do not refer to the treatment centers or refer late. Physicians do not pay attention to the differential diagnosis of tuberculosis, and their main focus is on the diagnosis and treatment of COVID-19.”*

The results of the literature review about the effect of health insurance on the medication adherence of elderly patients with tuberculosis were contradictory [66, 67, 81]; however, all the interviewed groups pointed to the positive role of insurance in medication adherence.

## 4. Discussion

This qualitative study was first conducted to identify the factors affecting the medication adherence of elderly tuberculosis patients in Iran. The results of this study showed that medication adherence in elderly tuberculosis patients is a complex and multidimensional phenomenon with the related factors that are placed in four main categories of individual factors, interpersonal factors, factors related to healthcare service provider centers, and extraorganizational factors.

Based on the findings of the present study, the most effective factors in medication adherence were placed in the main category of individual factors and subcategories of biological factors, affective-emotional factors, behavioral factors, cognitive factors, factors related to tuberculosis, and economic factors. The results of the present study suggested a relationship between the age of elderly tuberculosis patients and medication adherence; accordingly, elderly patients with older age had less medication adherence. Kalhori's study in Iran showed that the probability of failure in tuberculosis treatment was higher in the elderly [86]. Medication nonadherence is one of the main factors in failure in tuberculosis treatment [2, 14]. Therefore, it is important to pay special attention to elderly patients with tuberculosis in countries, such as Iran.

The results of this study showed that the presence of comorbidity reduced medication adherence in elderly tuberculosis patients. Aging accompanies comorbidity, increased required medications and more complex treatment regimens [42], and drug interactions due to polypharmacy [8]. Consequently, the presence of comorbidity can also affect the continuation of the antituberculosis treatment [87].

The results of the present study showed that affective and emotional factors influenced the medication adherence of elderly tuberculosis patients. In addition, the findings of the review section of the present study indicated less medication adherence in elderly tuberculosis patients and depression. Depression is a common disorder in patients with tuberculosis that causes adverse treatment outcomes [88, 89]. In a study, Koyanagi et al. depicted that depression negatively affected the self-care process in patients with tuberculosis [89].

In this study, the effect of treatment response on medication adherence was inconsistent. In general, about 2 weeks after starting the treatment, the symptoms of numerous patients improved. Therefore, symptom improvement might be an obstacle to continue the treatment [70]. In a study on nonelderly patients with tuberculosis, feeling better as a result of treatment was also related to medication nonadherence [90].

Side effects due to tuberculosis medications are among other obstacles to continue the treatment [91]. Physiological changes, comorbidity, polypharmacy, and the possibility of side effects caused by tuberculosis medications increase by age [8]. In various studies, the side effects of tuberculosis medications were higher in the elderly [92–95]. Therefore, in the treatment of an elderly individual with tuberculosis, the possibility of showing side effects and their correct management should always be considered.

In this study, poverty, the financial burden caused by treatment, and loss of job and occupational opportunities were among the factors of medication nonadherence in elderly tuberculosis patients. Various studies have shown that tuberculosis is related to poverty [90, 96]. Poverty is an obstacle to the successful implementation of tuberculosis control programs [97]. Poor individuals, especially the elderly in the developing countries, face more obstacles for receiving healthcare and treatment [18, 97]. In Carlsson's et al. qualitative study, nurses mentioned poverty and lack of adequate nutrition as the reasons for discontinuing tuberculosis treatment [98]. In Gebreweld's et al. study, losing a job and the resulting financial problems following the diagnosis of tuberculosis in the elderly are associated with nonadherence to medication [78].

The results of this study demonstrated that the relationship of elderly tuberculosis patients with the treatment team and family members affected medication adherence. The proper relationship between the elderly patient and the members of the treatment team (i.e., physicians and nurses) is an essential principle in disease management and treatment. Moreover, providing sufficient information and educating patients are necessary for proper communication, which affects medication adherence [99]. Studies conducted on individuals with chronic diseases also showed the effect of the relationship between the patient and the physician on medication adherence [100]. In a study on the elderly with a disease other than tuberculosis, Ben-Natan and Noselozich showed a positive relationship between the patient's trust in physician's professional ability and their medication adherence [101].

Tuberculosis patients need proper education regarding tuberculosis treatment [102]. However, the results of the present study showed that according to some physicians and nurses, providing more information to elderly tuberculosis patients made these patients confused and worried; as a result, they did not provide the patients with enough information. Various studies also showed the presence of a negative attitude toward the elderly in healthcare environments. Ageist stereotypes, judgments, and discrimination are important barriers to health equity, in addition to the quantity and quality of care provided for the elderly. Therefore, it should be noted that discrimination based on age affects the clinical practice and decision-making of healthcare providers [103].

The health culture of tuberculosis patients is affected by the information received from family, friends, neighbors, and other members of society [104]. Although no specific study was carried out on the elderly in this regard, the results of other studies revealed the effect of family relationships on the medication adherence of patients with tuberculosis [105, 106]. In a study on nonelderly patients with tuberculosis, Liefooghe and Muynck also suggested that one of the principles of successful treatment of tuberculosis in the intensive phase was encouraging the patient to continue treatment by family members [105].

The results of this study indicated the influence of factors related to healthcare service provider centers on the medication adherence of elderly tuberculosis patients. To date, no study has been conducted on the effect of the aforementioned factors on the medication adherence of elderly tuberculosis patients; nevertheless, the results of the present study showed that the medical centers' facilities and capacity building in healthcare service provider, including motivating the medical personnel, play an important role in the medication adherence of elderly tuberculosis patients. The results of Dimitrova's et al. study on nonelderly patients with tuberculosis also showed that healthcare workers' insufficient salary and lack of motivation are among important obstacles to tuberculosis treatment adherence [107].

The results of the present study illustrated that extraorganizational factors, including social factors and health policymaking, also affected the medication adherence of elderly tuberculosis patients. Based on the findings of this study, the culture of society is one of the important and influential social factors in the medication adherence of elderly individuals with tuberculosis. One of the important cultural factors is the stigma caused by tuberculosis. Stigma is one of the obstacles to the timely referral of the patient to the treatment centers, and as a result of treatment discontinuation, which has been mentioned in various studies, has negative effects on medication adherence [107–111]. Tuberculosis is an infectious and threatening disease. Therefore, there are widespread negative perceptions about this disease. Most individuals consider tuberculosis a social disease and limited to specific population groups with antisocial behaviors [107]. In Woith's and Rappleyea study, negative feelings about tuberculosis were related to stigma, and the elderly had more negative feelings about tuberculosis [112].

In the current study, the use of traditional treatments was another factor influencing the medication adherence of elderly individuals with tuberculosis. In other studies, patients' turning to traditional tuberculosis treatments was also related to medication nonadherence [30, 91, 113]. The belief in the effectiveness of traditional medications, the shorter period of traditional treatments, and the lower costs of these types of medications are the reasons for patients to turn to traditional treatment [30]. In Gele's et al. study, difficult access to medical centers, especially in vulnerable patients such as the elderly, was one of the factors for choosing traditional treatments [113]. Therefore, in the treatment process of an elderly tuberculosis patient, the possibility of using traditional medications and the factors affecting this choice should always be taken into consideration.

The results of this study depicted the effect of social support on the medication adherence of elderly tuberculosis patients. In general, the elderly need social support more than other age groups [114], and those who live alone have more problems taking their medications [115]. In a study, Lu et al. suggested that the social isolation of elderly patients with chronic diseases had a negative effect on the social support and medication adherence of the elderly [116].

The findings of the present study indicated the effect of health insurance on the medication adherence of elderly tuberculosis patients. Dong's et al. study showed that using an insurance-based approach increased hospital admissions, outpatient visits, and drug use in poor patients with tuberculosis [97]. Since poverty, financial dependence of family members on pensions for the elderly, and lack of access to healthcare are common problems of the elderly, especially in the developing countries [18], it can be expected that paying special attention to health insurance coverage for elderly patients with tuberculosis is very essential to promote medication adherence in this age group.

There were some limitations for the present study due to the impossibility of access to some relevant texts, especially full texts, and the need for English studies. The study sampling was performed during the COVID-19 pandemic, which limited the interviews, although it was tried to conduct the interviews by following the protocols and through phone calls, if necessary. One of the strengths of the present study is the integrative literature review. The integrative literature review not only brings together scattered data and creates a basis for conducting specific studies on the elderly but also provides the possibility of entering studies with quantitative and qualitative methods to the review [43] and develops a comprehensive understanding of the problems related to healthcare and health policymaking [43]. Another strength of the current study is conducting individual interviews with physicians, nurses, elderly tuberculosis patients, and their family caregivers. This part of the study led to a better and deeper understanding of the factors affecting medication adherence in elderly tuberculosis patients.

## 5. Conclusion

Despite numerous attempts, a large number of tuberculosis patients, especially the elderly, have not yet had complete medication adherence. Therefore, the identification of the factors related to medication adherence and implementation of appropriate interventions in this age group will be an important step towards maintaining and improving the health of tuberculosis patients and other members of the society. The results of the present qualitative study illustrated that medication adherence in elderly tuberculosis patients was not limited to physical and biological factors; however, it was a multidimensional phenomenon and was influenced by a wide range of individual factors, interpersonal factors, factors related to healthcare service provider centers, and extraorganizational factors. Therefore, it is necessary for society, policymakers, and healthcare providers to have a more comprehensive look at the factors affecting medication adherence in this group of patients to plan and implement more effective interventions.

## Figures and Tables

**Table 1:** Characteristics of participants in interviews.

Total number of participants	Number of participants in each group	Job/number		*Gender*		Age range (year)	Interviewed group	Participant's number
				Male	Female			
20	10	2	Nurse	1	9	35–50	Healthcare professionals	1–10
		5	General physician					
		3	Pulmonologist					
	6	2	Housewife	4	2	67–86	Elderly patients with tuberculosis	11–16
		1	Retired					
		3	Manual worker					
	4	3	Housewife	1	3	24–60	Caregivers	17–20
		1	Employee					

**Table 2:** Categories and subcategories obtained from the integrated literature review and holding interviews.

Main categories	Subcategories	Primary subcategories
Individual factors	Biological factors	Age
		Gender
		Comorbidity
		Polypharmacy
		Activities of daily living (ADL)
		Instrumental activities of daily living (IADL)
	Affective-emotional factors	Motivation for treatment
		Depression and anxiety
		Patient's affection and feelings
		Patient's personality
		Acceptance of illness
	Behavioral factors	Patient's self-care ability
		Substance abuse
		Use of reminders for taking medication
	Cognitive factors	Effective interaction with the physician
		Memory
		Patient's knowledge about tuberculosis and treatment
		Patient's general knowledge
	Factors related to tuberculosis	Type of tuberculosis
		Characteristics of pharmacotherapy
		Response to treatment
		Medication side effects
	Economic factors	Patient's financial ability
		Patient's employment

Interpersonal factors	Relationship with the treatment team	Professional ethics of the treatment team
		Professional competence of the treatment team
		Trust in the treatment team
		Educating the patient and caregiver by the treatment team
	Family-related factors	Supportive family
		General characteristics of the family caregiver
		Family caregiver knowledge

Factors related to healthcare service provider centers	Medical centers' facilities	Responding to the patients' needs
		Centers' equipment
		Resource adequacy
	Capacity building in healthcare service provider	Educating the staff
		Monitoring the health of staff
		Motivating the staff

Extraorganizational factors	Social factors	Tuberculosis stigma
		Belief in traditional treatment
		Social development level
		Social support
	Health policymaking	Existing treatment protocols
		Level of insurance coverage

## Data Availability

The data are available and there is no restriction on additional information.
